# Exosomal miRNA-146a-5p Derived from Senescent Hepatocellular Carcinoma Cells Promotes Aging and Inhibits Aerobic Glycolysis in Liver Cells via Targeting IRF7

**DOI:** 10.7150/jca.96500

**Published:** 2024-06-17

**Authors:** Sijia Yang, Ang Li, Lihong Lv, Zhihua Zheng, Peiqing Liu, Jun Min, Jinxing Wei

**Affiliations:** 1 Guangdong Provincial Key Laboratory of Malignant Tumor Epigenetics and Gene Regulation, Sun Yat-sen Memorial Hospital, Sun Yat-sen University, 510120 Guangzhou, Guangdong, China.; 2 Department of Hepatobiliary Surgery, Sun Yat-sen Memorial Hospital, Sun Yat-sen University, 510120 Guangzhou, Guangdong, China.; 3 Clinical Trial Institution of Pharmaceuticals, Sun Yat-sen Memorial Hospital, Sun Yat-sen University, 510120 Guangzhou, Guangdong, China.; 4 Guangdong Provincial Key Laboratory of New Drug Design and Evaluation, Guangdong Province Engineering Laboratoty for Druggability and New Drug Evaluation, School of Pharmaceutical Sciences, Sun Yat-sen University, Guangzhou 510006, PR China.

**Keywords:** hepatocellular carcinoma, senescence, exosome, miRNA-146a-5p, IRF7, aerobic glycolysis, CHK2

## Abstract

Hepatocellular carcinoma (HCC) is a major global health challenge. Chemotherapy can cause HCC cells to become senescent. Senescent HCC cells play an important role in inhibiting or promoting cancer by producing extracellular vesicles with a senescence-associated secretory phenotype (EV-SASP). miRNA can be strongly upregulated in EV-SASP during the aging process and can substantially alter the phenotypic characteristics of cells. MiRNA microarray analysis revealed that miRNA-146a-5p was highly expressed in oxaliplatin- and H_2_O_2_-induced senescent Huh7 cells, and RT‒PCR confirmed its significant upregulation in exosomes. The transcriptome sequencing results of Huh7 cells overexpressing miRNA-146a-5p suggested that miRNA-146a-5p could regulate HCC cell glycolysis. Subsequently, a dual luciferase assay was used to verify whether miRNA-146a-5p can interact with IRF7 to promote aging. The key functions of miRNA-146a-5p and IRF7 in aerobic glycolysis in liver cancer cells were determined through experiments analyzing glucose uptake, lactate production, the oxygen consumption rate (OCR) and the proton efflux rate (PER). Subsequently, the regulatory effect of IRF7 on the key glycolytic gene PFKL was confirmed through luciferase reporter assays. The western blot experiment results showed that miR-146a-5p can activate CHK2 and p53 phosphorylated proteins by targeting IRF7, and upregulate p21 protein. Overexpression of miRNA-146a-5p effectively inhibited the aerobic glycolytic function of HCC cells. Moreover, silencing IRF7 effectively inhibited aerobic glycolysis. MiR-146a-5p. MiR-146a-5p can activate the phosphorylation of CHK2 phosphorylation protein and its downstream protein p53 by targeting IRF7, and the activated p53 upregulates the expression of p21. Our study revealed that exosomal miRNA-146a-5p produced by aging HCC cells, can inhibit HCC cell proliferation through inhibiting aerobic glycolysis and promote HCC cell aging by activating CHK2/p53/p21 signaling way by targeting IRF7.

## Introduction

Ninety percent of cases of primary liver cancer are hepatocellular carcinoma (HCC), which is also the fourth most common cause of cancer-related fatalities globally, accounting for hundreds of thousands of deaths every year. It is estimated that by 2025, more than one million persons will be affected with HCC per year. Although there are several available treatment methods for HCC, patients diagnosed with advanced HCC often have a poor prognosis^1^-^3^.

Cellular senescence is a process by which cells age and permanently stop proliferating, and senescence has inhibitory effects on tumor cells. In general, there are two types of senescence related to cancer: oncogene-induced senescence (OIS) and treatment-induced senescence (TIS)^4^-^6^. Senescence-inducing cancer cells may be a viable treatment approach, according to mounting data during the last ten years^7^. It has been demonstrated that p53-dependent or p53-independent signaling can regulate cellular senescence in HCC^8^,^9^. Furthermore, if senescence is suppressed, malignant transformation can still occur in cirrhotic liver tissue with a large fraction of senescent liver cells. Treatment for HCC might therefore benefit from investigating the variety of senescence-associated processes in HCC.

Exosomes are membrane-bound extracellular vesicles that are formed in both normal and pathological situations in the inner compartments of the majority of eukaryotic cells^10^. Extracellular vesicles, which contain proteins, miRNAs, mRNAs, DNA, and lipids, play important roles in local and long-distance intracellular communication^11^. Moreover, miRNAs are small noncoding RNAs that act as oncogenes or tumor suppressors in different types of cancer^12^. At present, there is still a lack of research on the mechanism by which exosomal miRNAs produced by aging cells participate in cellular communication during the development of liver cancer.

In this work, we examined the expression patterns and predictive significance of miRNA-146a-5p and its target gene, IRF7, in senescent HCC cells treated with hydrogen peroxide and oxaliplatin. The exosomes of aged HCC cells showed a substantial upregulation of miRNA-146a-5p. Furthermore, miRNA-146a-5p inhibited the glycolytic function of HCC cells, which accelerated aging and reduced cell proliferation. We discovered that IRF7, one of miRNA-146a-5p's target genes, stimulates the expression of key glycolytic gene PFKL via direct transcriptional regulation and prevents HCC cells from aging via inhibiting CHK2/p53/p21 pathway. Furthermore, we showed that IRF7 functions as a transcription factor to increase the expression of PFKL, a crucial glycolytic factor.

## Materials and methods

### Cell culture and tissue collection

The human HCC cell lines HepG2, Huh-7, and MHCC-97H were grown in DMEM with 10% fetal bovine serum added as a supplement. At Sun Yat-Sen University's Sun Yat-Sen Memorial Hospital, blood and tissue samples were taken from twenty HCC patients. Each research subject gave written, informed consent.

### Induction of HCC cell senescence

H_2_O_2_ and oxaliplatin were used to induce HCC cell senescence. Cells at 80% confluence were exposed to different doses of H_2_O_2_ (0, 25, 50, 100, or 150 µM) or oxaliplatin (0, 4, 8, or 16 µM) for 24 h to 48 h and then washed with PBS, after which the media was changed to fresh media.

### SA-β-Gal staining analysis

HCC cells and mouse tissue sections were stained using a senescence-related factor β-galactosidase (SA-β-Gal) staining kit from Beyotime (Shanghai, China). The number of stained cells was counted under a microscope.

### Cell transfection

HCC cells were transfected with miR-146-5p mimic (50 nmol/L), miR-146 inhibitor, or their corresponding negative controls in 6-well plates containing roughly 5×10^5^ cells per well. For effective transfection, JetPRIME® (Polyplus-transfection S.A., France) was added to the medium concurrently. The transfection reagent was withdrawn and new culture media was introduced after six hours. Twenty-four hours after transfection, selection was done. The miR-146a mimic, miR-146a-5p inhibitor and their negative controls were purchased from RiboBio (China). The wild-type and mutant plasmids of IRF7 were constructed using pmirGLO (Guangzhou Jetway Biotech, China) as the vector, and the HCC cells were transfected using the same reagents and methods mentioned above.

### Exosome extraction, identification, and uptake

For 72 hours, HCC cells were grown in 10-cm dishes using vesicle-depleted media. An exosome separation kit for cell culture media (Meilunbio, China) was used to remove the exosomes from the culture medium after it had been collected. Using an ExoQuick Plasma Prep and Exosome Precipitation Kit (System Biosciences, USA) and following the manufacturer's instructions, circulating exosomes were separated from plasma. Transmission electron microscopy (TEM) (JEM-1400, JEOL, Japan), Western blot analysis, and nanoparticle tracking analysis (NTA) (Particle Metrix, Germany) were used to identify exosomes. The uptake of exosomes by HCC cells was observed by PKH26 staining (Sigma, USA). HCC cell suspensions were seeded in a confocal plate at 5×10^4^ cells/well. Following the cells' attachment to the well bottoms, the plate was incubated for 24 hours with the prestained exosome suspension added, containing 100 μg/mL of protein. We used a Leica confocal microscope to capture the images.

### Flow cytometry analysis

HCC cells were harvested and labeled with PI and Annexin V-FITC (Pricella, China), and flow cytometry and FlowJo software were used to analyze apoptosis. After being fixed in 70% ethanol for two hours, HCC cells were stained with PI (Beyotime, China), and then, cell cycle analysis was performed with ModFit and flow cytometry.

### Real-time PCR (qRT‒PCR)

TRIzol reagent (TaKaRa, China) was used to extract total RNA, and a Roche LightCycler 480 instrument was used to quantify the result. Using a SYBR® Green Master Mix Kit (TOYOBO, Japan), qRT-PCR was carried out in accordance with the manufacturer's recommendations. The target genes' expression levels were standardized to match GAPDH's expression level. With the use of the comparative Ct method (ΔΔCt method), the relative expression was computed. Table S1 lists the primer sequences.

### Western blotting

Total proteins were extracted using lysis buffer (KeyGEN, China) in preparation for Western blotting. The Bradford assay (Thermo, USA) was used to calculate the protein concentration. After being separated using 10% SDS-PAGE, the protein samples were transferred to membranes made of polyvinylidene difluoride (Millipore, Immobilon, Ireland). Following an overnight incubation period of 4 °C for primary antibodies and 1 hour for secondary antibodies at 37 °C, protein signals were identified using an enhanced chemiluminescence (ECL) detection system (Amersham Imager 600 and Bio-Rad, USA) and Femto-Sensitive Plus ECL Solution (MIKX, China). Table S2 contains information about the specific antibodies employed in the Western blotting process.

### Dual-luciferase reporter gene assay

After being amplified from genomic DNA, the PFKL gene's promoter region was subcloned and added to the PGL 3-Basic luciferase reporter plasmid. Simultaneously, a PFKL promoter-mutant plasmid bearing the anticipated IRF7 binding site was created, subcloned, and introduced into the pGL3-Basic vector. Through sequencing, the wild-type (WT) and mutant constructs were verified. After transfection for 24 hours, the cells were seeded into a 6-well plate, lysed using cell lysis solution, and then moved to a white microplate. The firefly luciferase activity was then measured after adding the firefly luciferase reaction solution. The activity was assessed following the inoculation with the Renilla luciferase reaction solution. We used kits that we bought from Beyotime (China).

### Co-immunoprecipitation (Co-IP)

Cells were lysed in IP lysis buffer supplemented with protease and phosphatase inhibitors. Lysates were cleared by centrifugation at 12,000g for 15 minutes at 4°C, and the supernatant protein concentration was determined using a BCA Protein Assay Kit (Thermo Fisher Scientific). For each Co-IP experiment, 300 µg of total protein lysate was incubated with 2 µg of the antibody against the protein of interest or a control IgG overnight at 4°C with gentle rotation. Protein A/G Plus-Agarose beads (MCE, USA) were then added to each sample and incubated for an additional 2-4 hours at 4°C to capture the antibody-protein complexes. The beads were collected by centrifugation at 1,000g for 5 minutes at 4°C and washed four times with IP lysis buffer. Bound proteins were eluted by boiling the beads in 2x loading buffer for 5 minutes and then subjected to SDS-PAGE followed by Western blot analysis using specific antibodies to detect the protein of interest and its interacting partners.

### Immunofluorescence

Cells underwent fixation in 4% paraformaldehyde for a duration of 15 minutes, followed by permeabilization with 0.1% Triton X-100 for 15 minutes. Subsequent to these treatments, nonspecific binding sites were blocked using 10% goat serum for 60 minutes. Thereafter, the specimens were incubated with a primary antibody directed against IRF7 and p21 at 4°C in a humidified chamber overnight. This step was succeeded by incubation with a fluorophore-conjugated secondary antibody for 60 minutes at ambient temperature. Fluorescence images were acquired utilizing a laser scanning confocal microscope, ensuring optimal resolution and specificity of the antigen-antibody interactions observed.

### Cell proliferation analysis

After two weeks of incubation, fixed cells were stained with 0.1% crystal violet for colony formation assays. For the purposes of the computation, colonies with more than 50 cells were included. A kit purchased from Beyotime Biotechnology (Shanghai, China) was used to conduct EdU tests. ImageJ was used to count the number of cells that were EdU-positive.

### Wound-healing assay

HCC cells were cultured until a monolayer of confluence formed. The monolayer was subsequently scraped using a 10-µl pipette tip. After that, NAM solution (10, 20, and 50 mM) was applied to the cells. Every 24 hours, the migratory distance was measured and photos were taken with a microscope.

### Migration and invasion assays

HCC cells were placed in the top Transwell chamber (pore size 12 μm, BD, USA), while 600 μl of DMEM supplemented with 15% FBS was placed in the bottom Transwell chamber. The cells that moved to the bottom chamber during the course of a 24-hour incubation period were fixed, stained, and then counted.

### Glucose and lactate assays

HCC cells transfected with si-RNA, the IRF7 overexpression plasmid, mimic-146a-5p, or inhibitor-146a-5p were sown at a density of 5×10^6^ cells/6 cm dish. The culture media or cells were taken out after a full day. Following the manufacturer's instructions, glucose absorption and lactate generation were measured using a glucose content assay kit (Geruisi, China) and a lactic acid content assay kit (Geruisi, China), respectively. For data normalization, total cell protein levels were utilized. The study was carried out in at least three duplicates.

### Intracellular ATP assay

HCC cells transfected with shRNA, the IRF7 overexpression plasmid, mimic-146a-5p, or inhibitor-146a-5p were sown at a density of 5×10^6^ cells/6 cm dish. Following a 24-hour period, the manufacturer's instructions for an ATP Assay Kit (Beyotime, S0026) were followed to determine the levels of ATP. For data normalization, total cell protein levels were utilized. The study was carried out in at least three duplicates.

### Seahorse Assay

Use the Seahorse XF 96 Extracellular Flux Analyzer (Agilent, USA) to determine the effects of miR-146a-5p and IRF7 on oxygen consumption rate and glycolysis rate in MHCC-97H and HepG2 cells. Inoculate cells into XF 96 cell culture plates to achieve 90% confluence, with 5000 cells per well. Various chemicals will be sequentially injected into each well and subjected to PER or OCR measurements. After the experiment was completed, the cells were immediately digested with trypsin and the single well rate data was standardized to protein concentration.

### Animal experiments

Animal experiments were conducted with the approval of the Ethics Committee of Sun Yat-sen University. Male thymus-free nude mice (BALB/c-nu/nu, 4 weeks old) were obtained from the Animal Center of Sun Yat-sen University. MHCC-97H cells transfected with the miRNA-146a-5p mimic or inhibitor (5 × 10^6^ cells in 100 μL of PBS) were subcutaneously injected into the right axilla of five nude mice in each group. Tumor volume was calculated as 0.5 × width^2^ × length and recorded every three days until the tumor volume reached 2000 mm^3^. After 4 weeks, the mice were anesthetized and sacrificed, and the tumors were excised, imaged, and weighed. The expression of p21, IRF7 and Ki67 in the tumor tissues was examined after they were fixed with 4% paraformaldehyde and incubated with antibodies.

### Statistical analysis

Statistical differences between the other two groups were analyzed using Student's t-tests, while statistical differences between means and repeated data from two or more groups were analyzed using ordinary one-way ANOVA with Tukey's multiple comparison tests. When analyzing two independent variables, each with two or more levels, ordinary two-way ANOVA with Šídák's multiple comparisons test was applied. To conduct statistical studies, GraphPad Prism (version 10) was used. Statistical significance was indicated by *P<0.05; **P<0.01; ***P<0.001; ****P<0.0001; ns P>0.05.

## Results

### Cellular senescent model conduction

**Figure 1:**
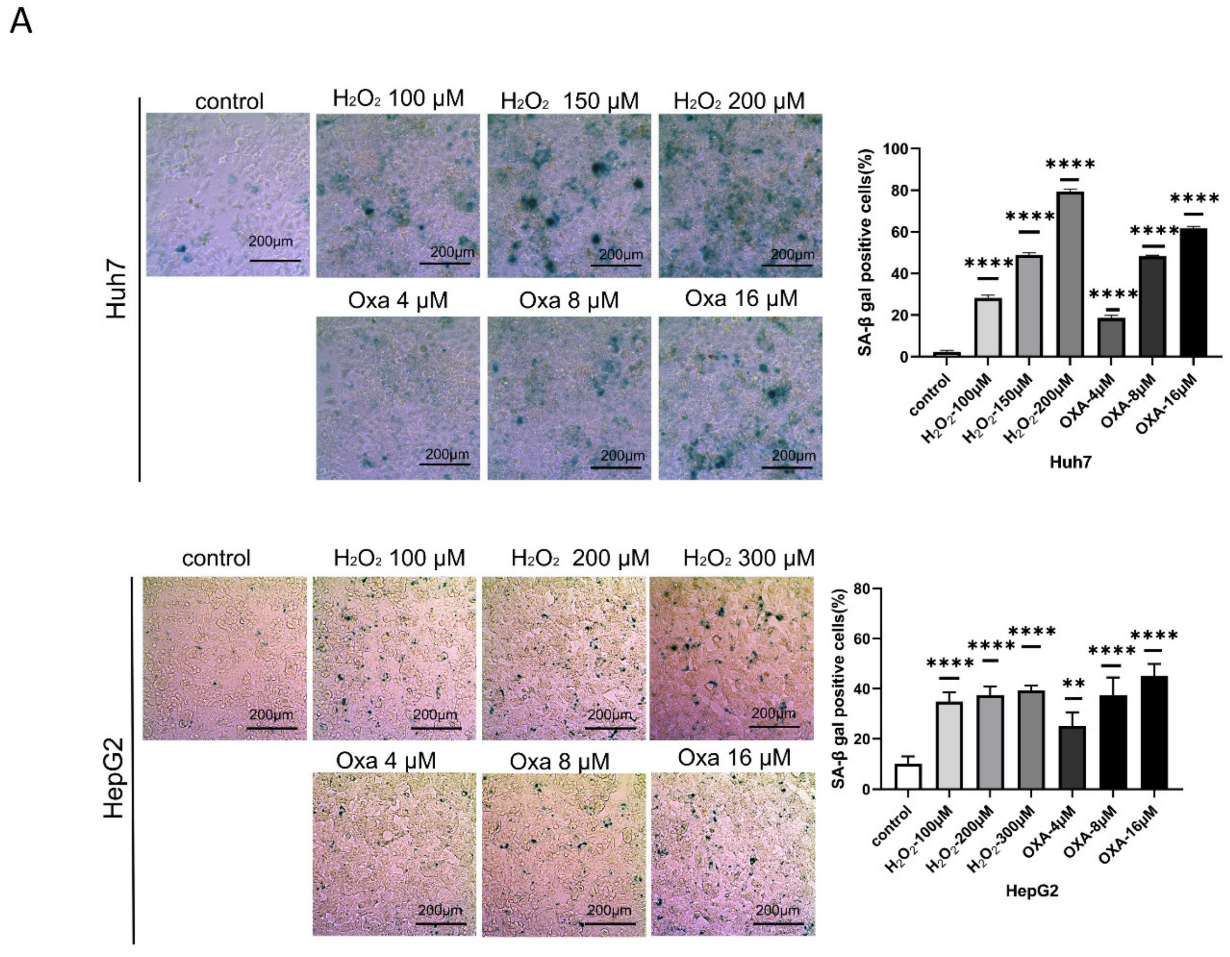

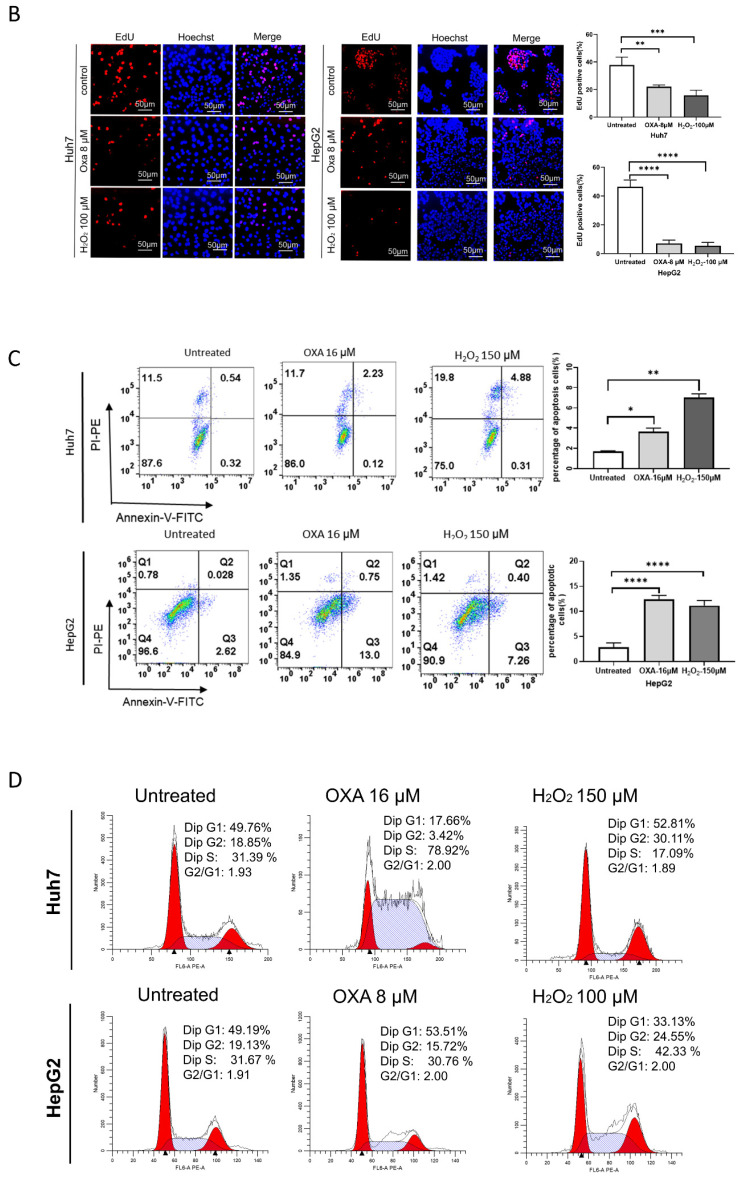
** Establishment of the cellular senescence model.** A. SA-β-gal staining of oxaliplatin- and H_2_O_2_-induced senescent Huh7 and HepG2 cells. B. Oxaliplatin- and H_2_O_2_-induced senescent Huh7 and HepG2 cell viability detected by an EdU assay. Apoptosis (C) and cell cycle distribution (D) of oxaliplatin- and H_2_O_2_-induced senescent Huh7 and HepG2 cells. OXA, oxaliplatin. H_2_O_2_, hydrogen peroxide.

We first calculated the efficiency of oxaliplatin and hydrogen peroxide (H_2_O_2_) in inducing senescence in Huh7 and HepG2 cells, respectively. Our data showed that treatment with either 16 μM oxaliplatin or 150 μM H_2_O_2_ for 24 h induced senescence in approximately 60% of HCC cells (Figure 1A-E). Therefore, we treated senescent HCC cells with oxaliplatin and H_2_O_2_ for 24 h.

### miRNA-146a-5p was overexpressed in the exosomes of senescent HCC cells

**Figure 2:**
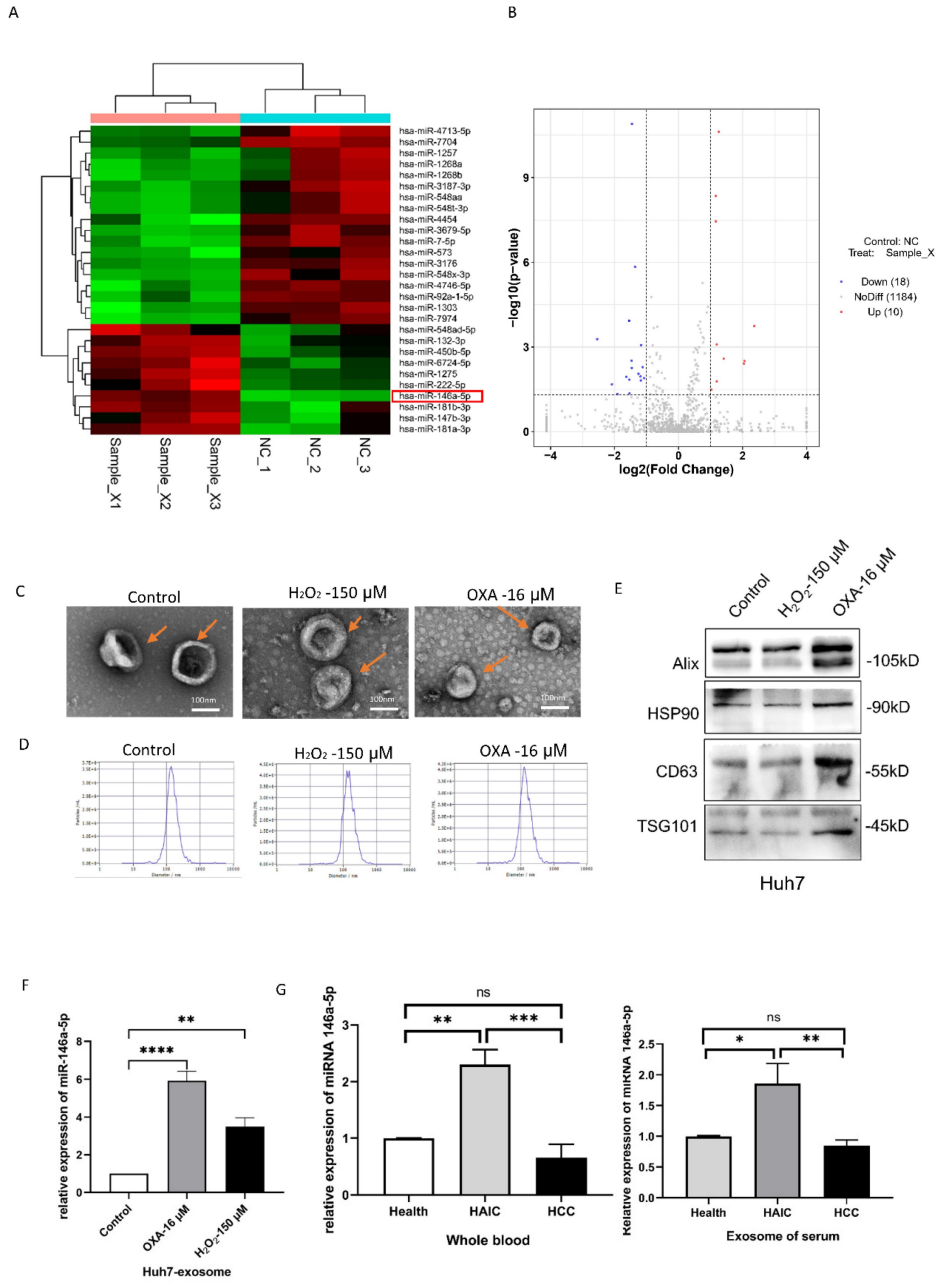
** miRNA-146a-5p was overexpressed in senescent HCC cells.** Heatmap (A) and volcano maps (B) of oxaliplatin groups and heatmap. X, oxaliplatin groups. C. Transmission electron microscopy images of exosomes isolated from the culture media of control Huh7 cells and oxaliplatin- and H_2_O_2_-induced senescent Huh7 cells (scale bar, 100 nm). D. Nanoparticle tracking analysis revealing the particle distribution of exosomes isolated from the culture media of control Huh7 cells and oxaliplatin- and H_2_O_2_-induced senescent Huh7 cells of various sizes. E. Representative Western blot images showing the biomarkers of exosomes isolated from the culture media of control Huh7 cells and oxaliplatin- and H_2_O_2_-induced senescent Huh7 cells, including Alix, HSP90, CD63 and TSG101. F. RT‒PCR assays were performed to determine the relative expression of miRNA-146a-5p in exosomes isolated from the culture media of Huh7 control cells and oxaliplatin- and H_2_O_2_-induced senescent Huh7 cells. G. The levels of miRNA-146a-5p in whole blood and plasma exosomes from 5 normal individuals, 5 HCC patients who received HAIC treatment before (HCC) and after (HAIC) were analyzed by real-time qPCR.

To determine the differentially expressed miRNAs in aging HCC cells, we first performed miRNA sequencing on aging Huh7 cells induced by 16 μM oxaliplatin and 150 µM H_2_O_2_. We found that oxaliplatin upregulated 560 miRNAs and downregulated 651 miRNAs in the aging group (Figure 2A-B). H_2_O_2_ upregulated 540 miRNAs and downregulated 688 miRNAs in the aging group. We found that the expression level of miRNA-146a-5p increased while the expression level of miRNA-7704 decreased in both the oxaliplatin- and H_2_O_2_-induced aging groups. The senescence of Huh7 cells was induced by oxaliplatin and H2O2, and exosomes were isolated from the supernatant of the cell culture medium using extracellular vesicle extraction agents. The exosomes were identified by transmission electron microscopy (TEM), nanoparticle tracking analysis (NTA), and Western blotting. TEM revealed that the exosomes had a typical cup or spherical shape (Figure 2C). According to the NTA results, the particle size distribution curve of the exosomes ranged from 55 to 200 nm (Figure 2D). All three groups of exosomes were enriched in exosomal protein markers such as Alix and CD63 (transmembrane/lipid binding protein), HSP90 (an important chaperone protein for protecting proteomic integrity), and TSG101 (a cytoplasmic protein) (Figure 2E). RT‒qPCR analysis confirmed that miRNA-146a-5p was significantly upregulated in the exosomes of aging Huh7 cells (Figure 2F). Compared with those in the whole blood and plasma exosomes of normal individuals and HCC patients who did not receive HAIC treatment, the levels of miRNA-146a-5p in the whole blood and plasma exosomes of HCC patients who received HAIC treatment was significantly greater (Figure 2G).

### Overexpression of miRNA-146a-5p induced senescence in HCC cells *in vitro*

**Figure 3:**
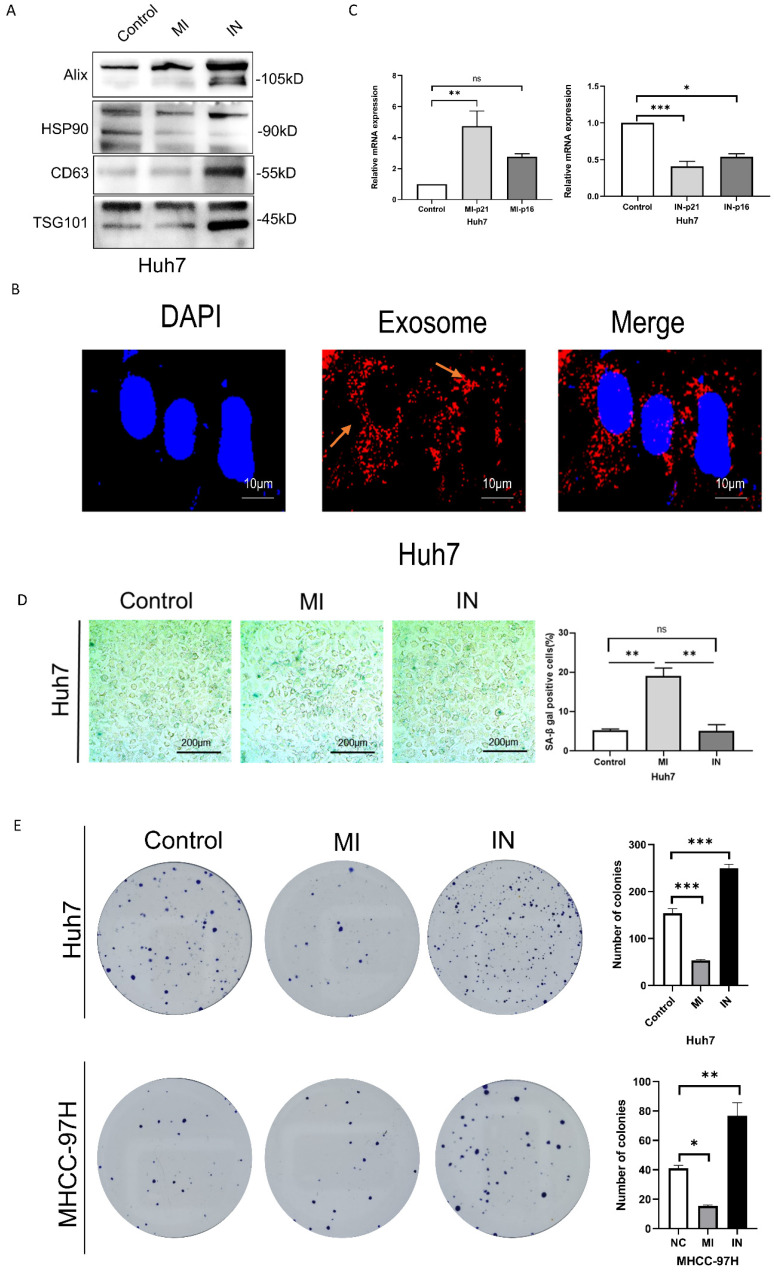

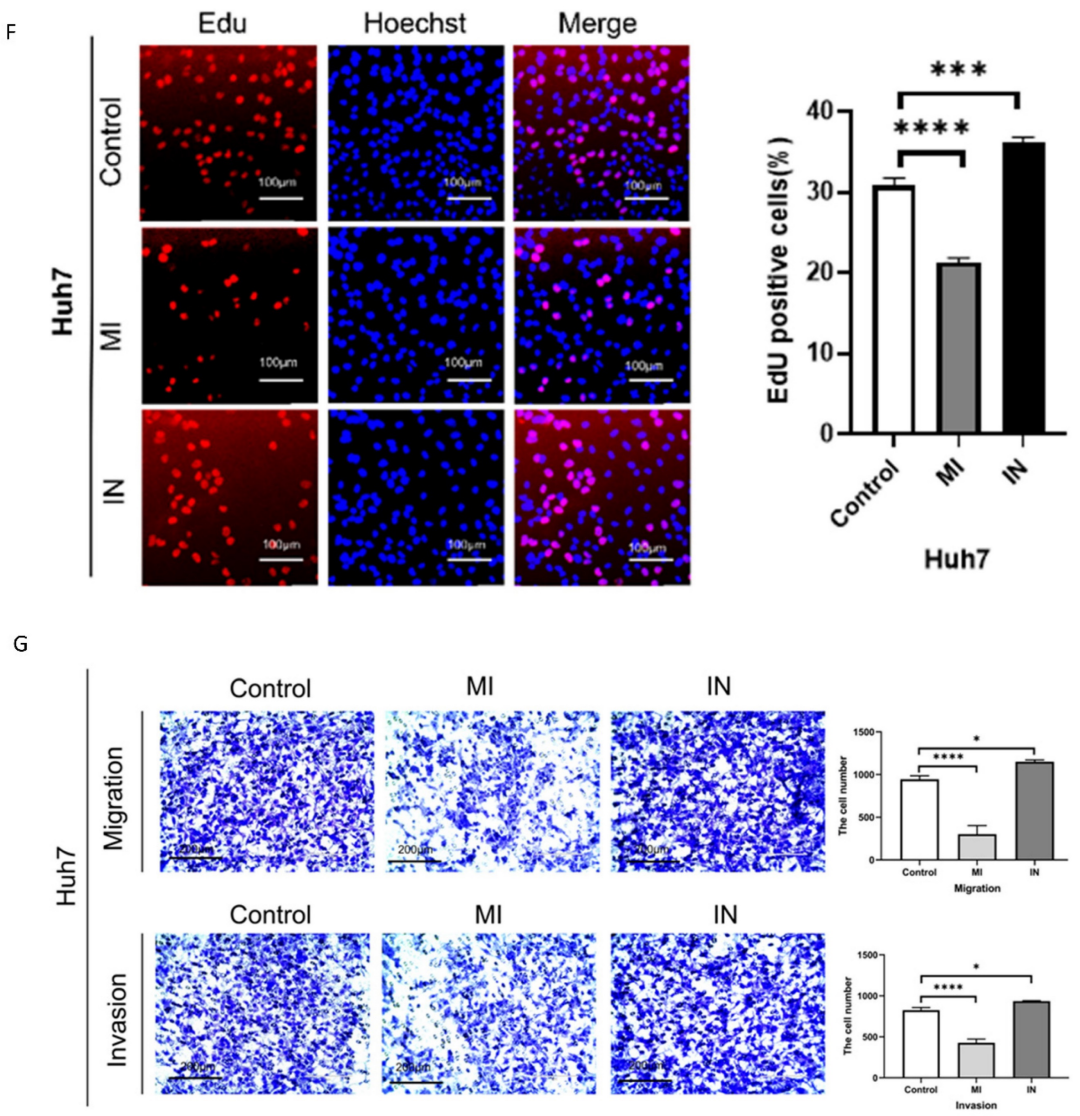
** Overexpression of miRNA-146a-5p induced senescence in HCC cells *in vitro*.** A. Extracellular vesicle markers were identified in Huh7 cells transfected with miRNA-146a-5p mimics and inhibitors using western blotting. B. For a predetermined amount of time, 200 ng/μl miRNA-146a-5p mimic exosomes labeled with L PKH26 were incubated with Huh7 cells. The cells were then examined under a fluorescent microscope (scale: 10 μm). C. Quantitative real-time PCR (qRT-PCR) of p21 and p16 senescence markers following co-culture of mimic-146a-5p exosomes or inhibitor-146a-5p exosomes with HCC cells. D. Sample photos of β-galactosidase (SA-β-gal) staining associated with senescence (scale bar, 200 μm). (E and F) After co-culture of mimic-146a-5p exosomes or inhibitor-146a-5p exosomes with HCC cells, the viability of HCC cells was assessed using colony formation (E) and EdU assays (F) with a 50 μm scale. G. Transwell assays verify the migration and invasion capabilities of Huh7 cells after co-culture with exosomes from mimic-146a-5p and inhibitor-146a-5p.

We used Western blotting to identify the exosomes of Huh7 cells transfected with miR-146a mimic or inhibitor (Figure 3A) and observed the uptake of extracellular vesicles by Huh7 cells after coculuter for 12 hours by PKH26 staining (Figure 3B). We conducted RT‒PCR, SA-β-gal staining and apoptosis experiments on HCC cells cocultured with exosomes containing miRNA-146a‒5p mimics or inhibitors and SA and confirmed that miRNA-146a‒5p promotes HCC cell aging. The RT‒PCR results showed that the mRNA levels of the aging marker p21 were significantly greater in the miRNA-146a-5p-overexpressing group, while the mRNA levels of p21 and p16 were significantly lower in the miRNA-146a-5p-inhibited group (Figure 3C). Overexpression of miRNA-146a-5p increased the percentage of SA-β-gal-positive cells in Huh7 cells (Figure 3D). Based on these findings, we determined the effect of miRNA-146a-5p overexpression on cellular aging. Then, we compared the proliferation and invasion abilities of HCC cells with and without miRNA-146a-5p transfection. Our data indicated that overexpression of miRNA-146a-5p significantly inhibited colony formation and proliferation in HCC cells (Figure 3E-F), while overexpression of miRNA-146a-5p significantly inhibited migration and invasion in HCC cells (Figure 3G). The impact of miR-146a 5p on the biological function of HCC cells indicates that miR-146a 5p plays an inhibitory role in the occurrence of HCC.

### IRF7 is a direct target of miR-146a-5p

**Figure 4:**
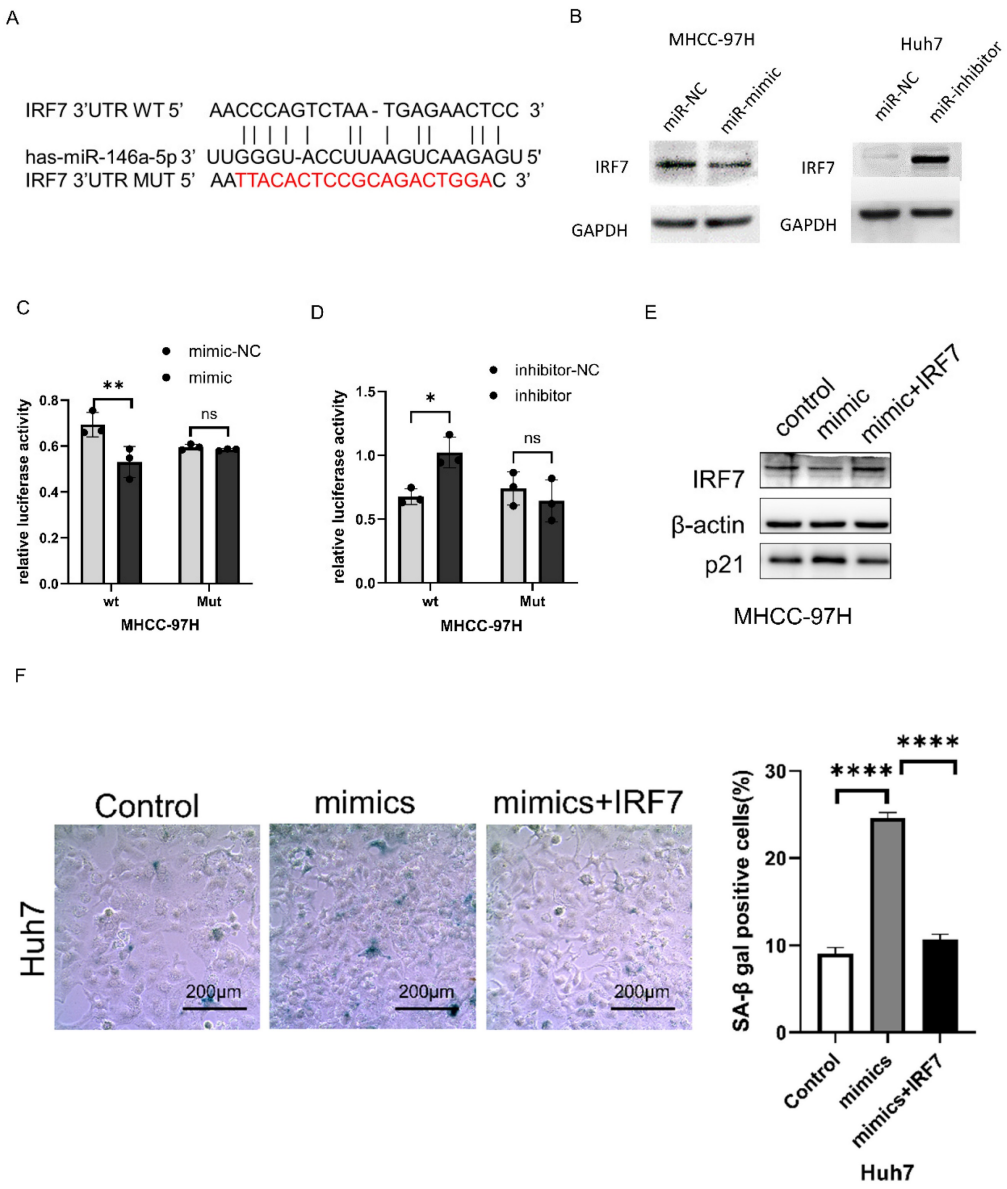
** IRF7 is a direct target of miR-146a-5p.** A. miRTarBase predicted the binding location of miR-146a-5p at the 3'UTR of IRF7. B. The impact of miR-146a-5p on IRF7 expression was assessed by Western blot analysis. C. Analysis was done on the luciferase activity in MHCC-97H cells co-transfected with NC or mimics together with pGL3-IRF7 or pmirGLO-IRF7Mut. D. Analysis was done on the luciferase activity in MHCC-97H cells that were co-transfected with inhibitors, NC, and either pmirGLO-IRF7 or pmirGLO-IRF7 Mut. (E) Protein immunoblotting experiments and (F)SA-β-gal assay confirmed IRF7 can reverse the pro-aging effects of miRNA-146a-5p.

Using miRTarBase (http://https://mirtarbase.cuhk.edu.cn/~MiRTarBase/miRTarBase_2022/php/index. php). IRF7 was identified as a potential target of miRNA-146a-5p (Figure 4A). Western blotting was used to detect changes in IRF7 expression after miRNA-146a-5p mimic and inhibitor transfection. As shown in Figure 4B, overexpression of miRNA-146a-5p reduced the expression of IRF7 in MHCC-97H cells, while inhibition of miRNA-146a-5p upregulated the expression of IRF7 in Huh7 cells. To verify that IRF7 is a direct target of miRNA-146a-5p, dual luciferase reporter gene assays were performed. We observed that co-transfection of MHCC-97H cells with miRNA-146a-5p mimics and the pGL3-IRF7 vector significantly decreased luciferase activity. Moreover, co-transfection of MHCC-97H cells with the miRNA-146a-5p inhibitor and the pGL3-IRF7 vector increased luciferase activity. However, in cells co-transfected with miRNA-146a-5p mimics or inhibitors and the pGL3-IRF7 mut vector, luciferase activity was not affected (Figure 4C-D). Protein blotting experiments and SA-β-gal staining assays confirmed that IRF7 can reverse the pro-aging effects of miRNA-146a-5p (Figure 4E-F). These results collectively demonstrate that IRF7 is a direct target of miRNA-146a-5p in the aging pathway.

### miR-146a-5p inhibits glycolysis

**Figure 5:**
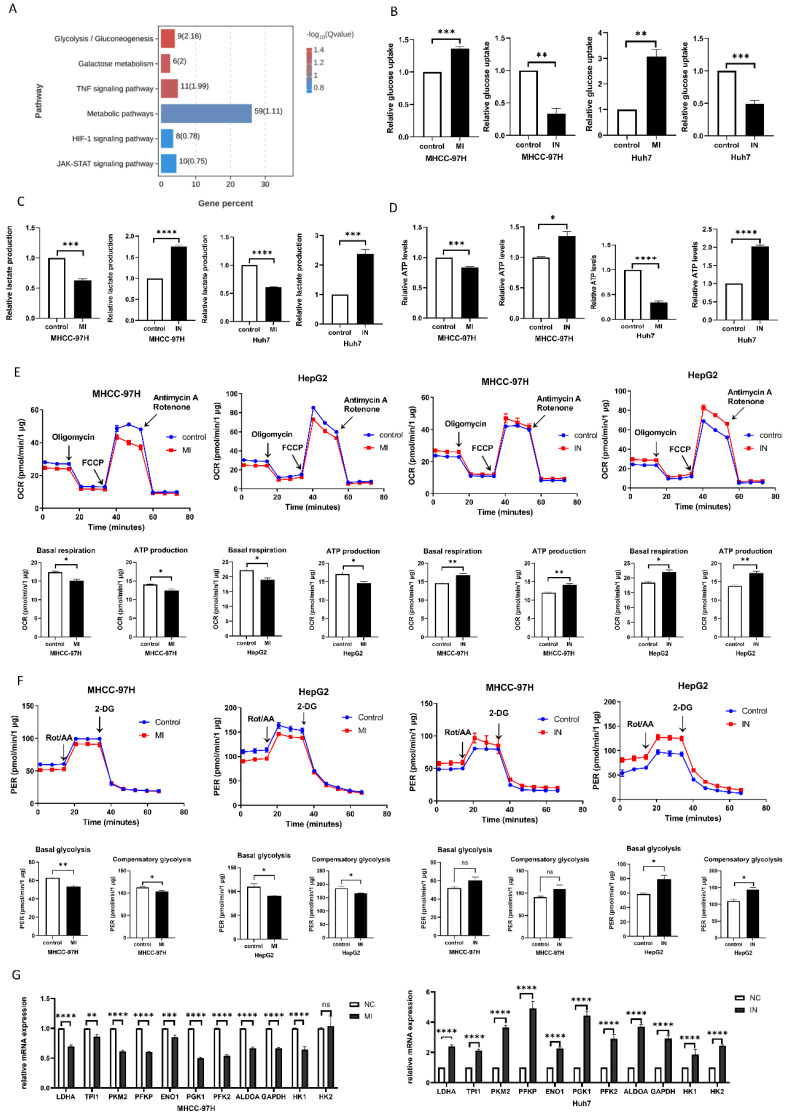
** miR-146a-5p inhibits glycolysis.** A. The functional enrichment analysis of miR-146a-5p and associated genes in HCC; (B-D) is validated by the transcriptome sequencing data. Experiments on glucose uptake (B), lactate production level (C), and ATP level (D). E. To assess ATP synthesis and basal respiration, the OCR was found. (F). As a measure of both basal and compensatory glycolytic flow, the PER was found. G. RT-PCR experiments validated the changes in relative mRNA expression levels of various glycolysis-related enzymes after co-culturing receptor HCC cells separately with exosomes from mimic-146a-5p and inhibitor-146a-5p. *P<0.05; **P<0.01; ***P<0.001; ****P<0.0001; ns P>0.05.

To verify the mechanism by which miRNA-146a-5p promotes aging in liver cancer cells, we performed transcriptome sequencing on Huh7 cells overexpressing and inhibiting miRNA-146a-5p. The sequencing results showed that miRNA-146a-5p is closely related to various metabolic pathways, such as the glycolysis/gluconeogenesis pathway, galactose metabolism, and other metabolic pathways closely related to aging (Figure 5A). To explore whether miRNA-146a-5p can affect glycolysis in HCC, we transfected miRNA-146a-5p into MHCC-97H and Huh7 cells and extracted the exosomes for coculture with HCC cells to measure glucose uptake and lactate production. We discovered that Huh7 and MHCC-97H cells' rates of glucose absorption were considerably enhanced by mimic-146a-5p and considerably decreased by inhibitor-146a-5p (Figure 5B).

**Figure 6:**
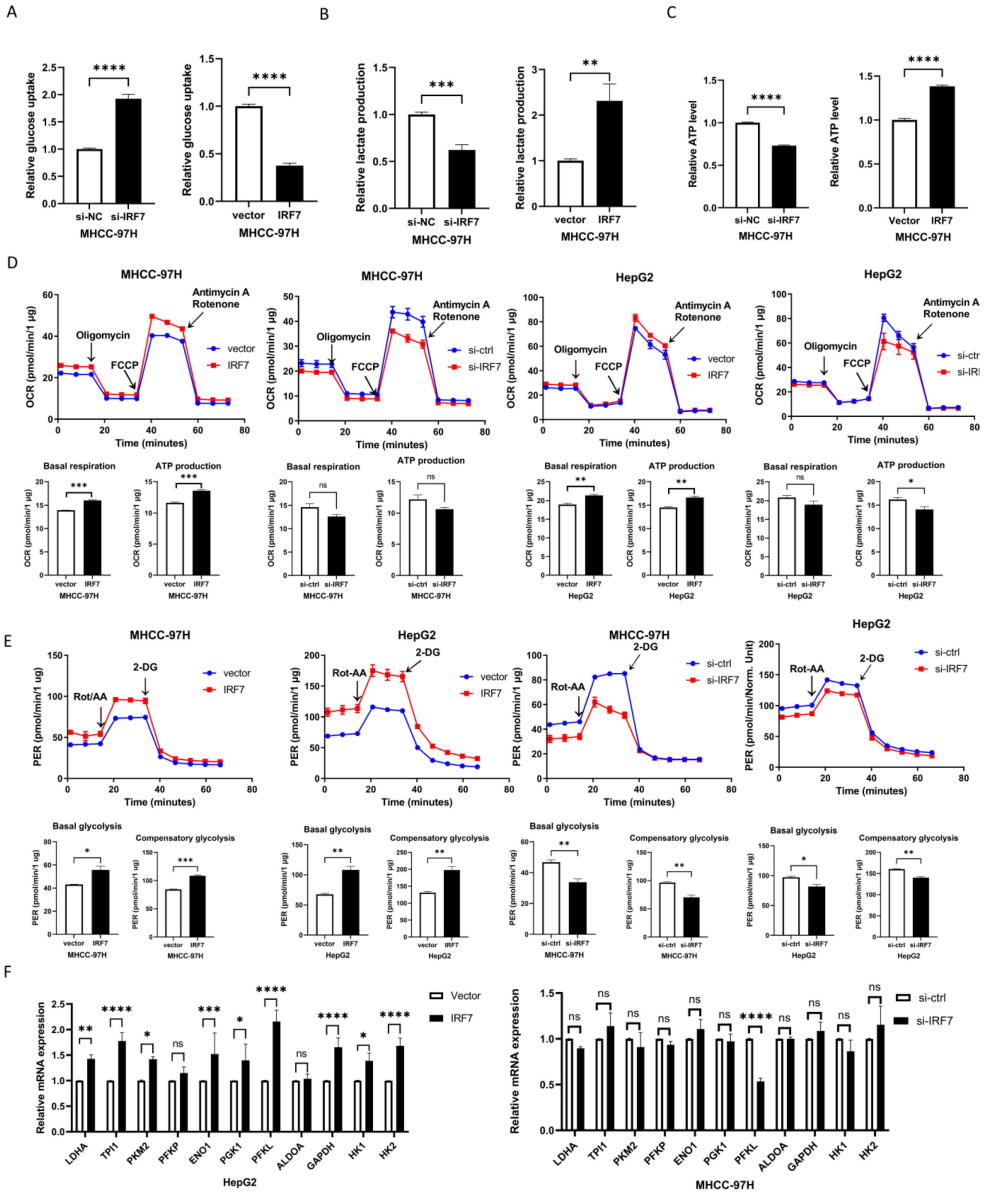

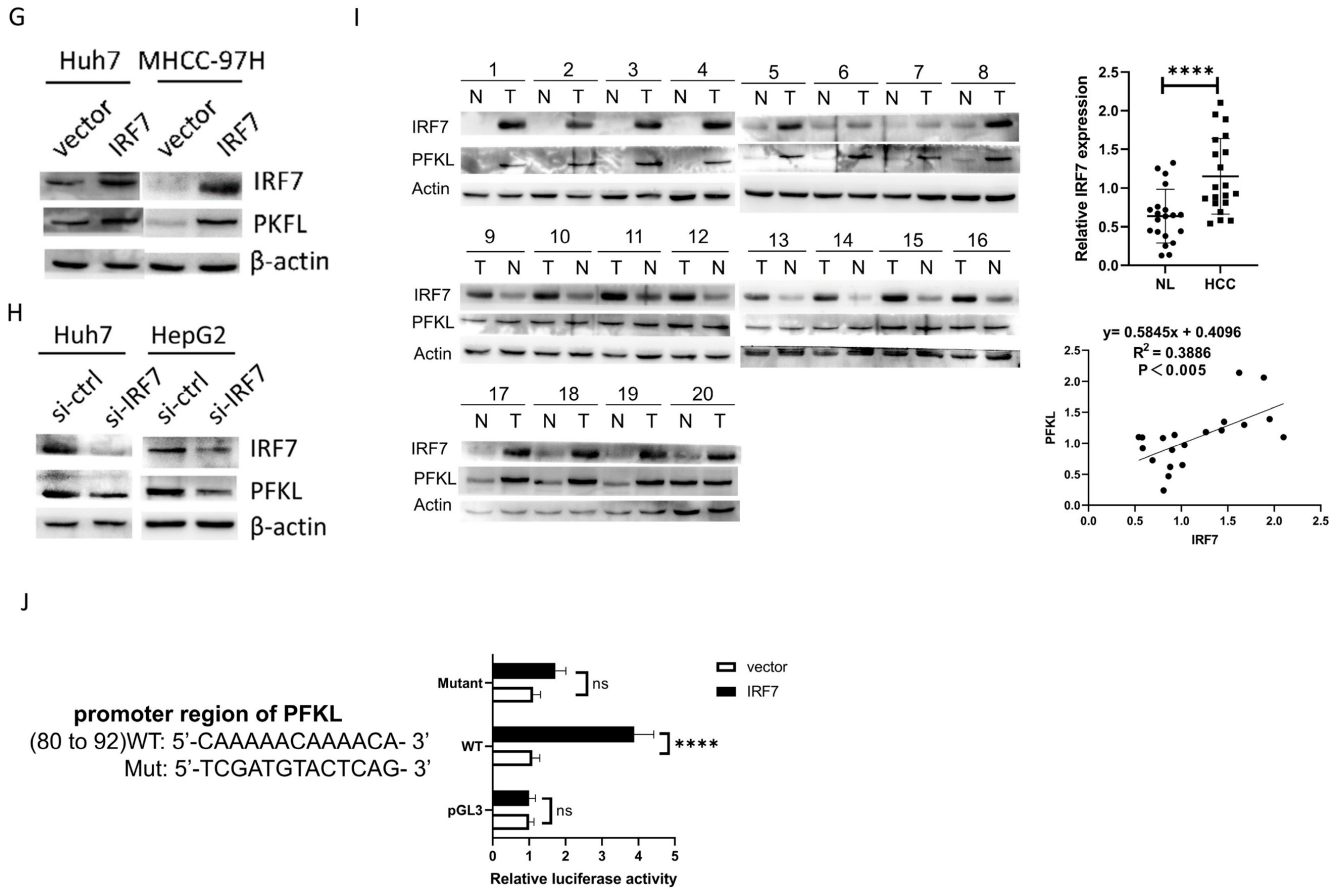
** IRF7 stimulates glycolysis by upregulating PFKL.** A. The impact of overexpressing IRF7 on the absorption of glucose (A), the synthesis of ATP (B), the secretion of lactate (C), the rate of oxygen consumption (D), and the rate of proton efflux (E). F. The impact of IRF7 overexpression on HepG2 and MHCC-97H cell glycolytic gene expression. (G) Western blotting was used to determine how IRF7 overexpression affected the expression of PFKL in Huh7 and MHCC-97H cells. (H) Western blotting was used to measure the impact of IRF7 silencing on PFKL expression in Huh7 and HepG2 cells. (I) Gene expression correlation study between PFKL and IRF7 in 20 patient samples with HCC. (J) PFKL gene promoter reporters' luciferase activity in Huh7 cells transfected with IRF7 or an empty vector. *P<0.05; **P<0.01; ***P<0.001; ****P<0.0001; ns P>0.05.

Additionally, we discovered that whereas inhibitor-146a-5p considerably increased lactate generation in Huh7 cells, mimic-146a-5p dramatically decreased lactate production in Huh7 cells (Figure 5C). The ATP levels in MHCC-97H cells were also measured, and the findings demonstrated that mimic-146a-5p dramatically decreased cellular ATP levels (Figure 5D). To further confirm this observation, we used a Seahorse XF-96 bioanalyzer to measure the proton efflux rate (PER) and oxygen consumption rate (OCR). As shown in Figure 6G, the coculture of miRNA-146a-5p exosomes with HCC cells resulted in PER and OCR decreases, indicating that miRNA-146a-5p inhibits the glycolytic ability of HCC cells. In contrast, cocultivation of inhibitor-146a-5p exosomes with HCC cells enhanced their glycolytic ability, as demonstrated by reduced glucose uptake, increased lactate production, elevated ATP levels, and elevated the PER and OCR (Figure 5E-F). Moreover, we further verified through PCR experiments that mimic-146a-5p can inhibit the mRNA levels of glycolytic enzymes related to the glycolis/gluconeogenesis pathway, such as HK1, HK2, PFKL, ALDOA, PGK1, ENO1, LDHA, PFKP, TPI1, PKM2, and GAPDH. This once again indicates that miRNA-146a-5p inhibits glycolysis (Figure 5G). In summary, miRNA-146a-5p inhibits glycolysis in HCC cells.

### IRF7 stimulates glycolysis by upregulating PFKL

In order to find out if IRF7, the target gene of miRNA-146a-5p, has an impact on glycolysis in HCC, we overexpressed IRF7 in Huh7 and MHCC-97H cells and assessed the amount of lactate produced and glucose taken up. The glucose absorption rate of Huh7 and MHCC-97H cells was found to be greatly reduced (Figure 6A) and lactate generation was elevated (Figure 6B) when IRF7 was overexpressed. Additionally, we discovered that HCC cells' ATP levels increased as a result of IRF7 overexpression (Figure 6C). We measured the OCR (Figure 6D) and (PER) (Figure 6E) using a Seahorse XF-96 bioanalyzer in order to further corroborate this finding. The glycolytic capacity of HCC cells is enhanced by IRF7 overexpression, as seen by decreased glucose uptake, increased lactate generation, elevated ATP levels, elevated PER, and elevated OCR. We examined the impact of IRF7 overexpression and inhibition on the expression of glycolytic genes in MHCC-97H cells using real-time qPCR analysis. Therefore, it can be concluded that PFKL may be a molecular transcriptional target of IRF7 in HCC cells because inhibition of IRF7 greatly downregulated PFKL in MHCC-97H cells whereas overexpression of IRF7 considerably upregulated PFKL (Figure 6F). Protein blot analysis showed that IRF7 silencing decreased PFKL protein levels in MHCC-97H and HepG2 cells (Figure 6H), while IRF7 overexpression increased PFKL expression in Huh7 and MHCC-97H cells (Figure 6G). IRF7 expression in clinical samples was positively correlated with PFKL expression in 20 HCC tissues (Figure 6I). We predicted the possible binding locations of IRF7 to JASPAR in the PFKL gene promoter in order to ascertain whether IRF7 can stimulate the transcription of PFKL. We discovered that IRF7 can considerably increase PFKL transcription in Huh7 and HepG2 cells using luciferase reporter gene assays (Figure 6J). Thus, by transcriptionally controlling the expression of PFKL in HCC, IRF7 could facilitate the glycolytic Walburg effect, according to the aforementioned studies.

### IRF7 promotes HCC cell proliferation and inhibits cell aging

**Figure 7:**
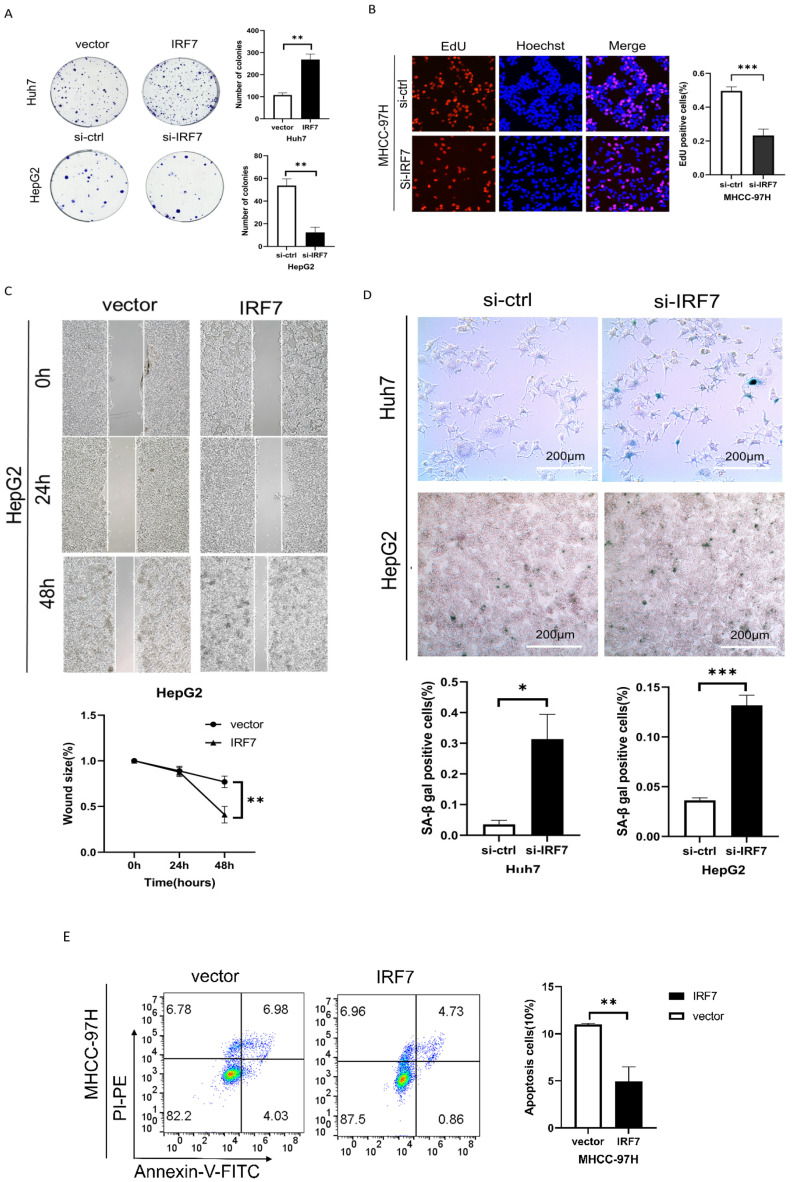
** IRF7 promotes HCC cell proliferation and inhibits cell apoptosis.** (A) The growth rate of HCC cells in which IRF7 was overexpressed or silenced in complete culture medium was determined through a plate colony formation assay. (B) The results of the EdU incorporation assay showed that IRF7 promoted the proliferation of MHCC-97H cells. (C) Measurement of wound healing in HepG2 cells overexpressing IRF7. (D) SA-β-gal staining of HCC cells with IRF7 overexpression or silencing. (E) Analysis of the effect of IRF7 overexpression on MHCC-97H cell apoptosis progression through flow cytometry. *P<0.05; **P<0.01; ***P<0.001; ****P<0.0001; ns P>0.05.

We performed plate colony formation and EdU tests to look into the impact of IRF7 on the growth of liver cancer cells. As seen in Figures 7A-7B, HCC cell growth was strongly stimulated by IRF7 overexpression, whereas HCC cell growth was suppressed by IRF7 knockdown. Wound healing experiments demonstrated that overexpressing IRF7 markedly increased the migratory capacity of HepG2 cells (Figure 7C). We investigated whether the target gene, IRF7, of miRNA-146a-5p had an aging-promoting effect on HCC cells. The number of SA-β-Gal-positive cells was considerably higher in the si-IRF7 group compared to the si-control group, as Figure 7D illustrates. The proportion of apoptotic cells in the IRF7 overexpression group was much lower than that in the empty vector group, according to the flow cytometry results (Figure 7E). These findings suggest that overexpression of IRF7 can promote the proliferation and migration of liver cancer cells, inhibit apoptosis of liver cancer cells, and also suppress the aging of liver cancer cells.

### miRNA-146a-5p promoted HCC cell senescence by upregulating members of the CHK2 signaling pathway

**Figure 8:**
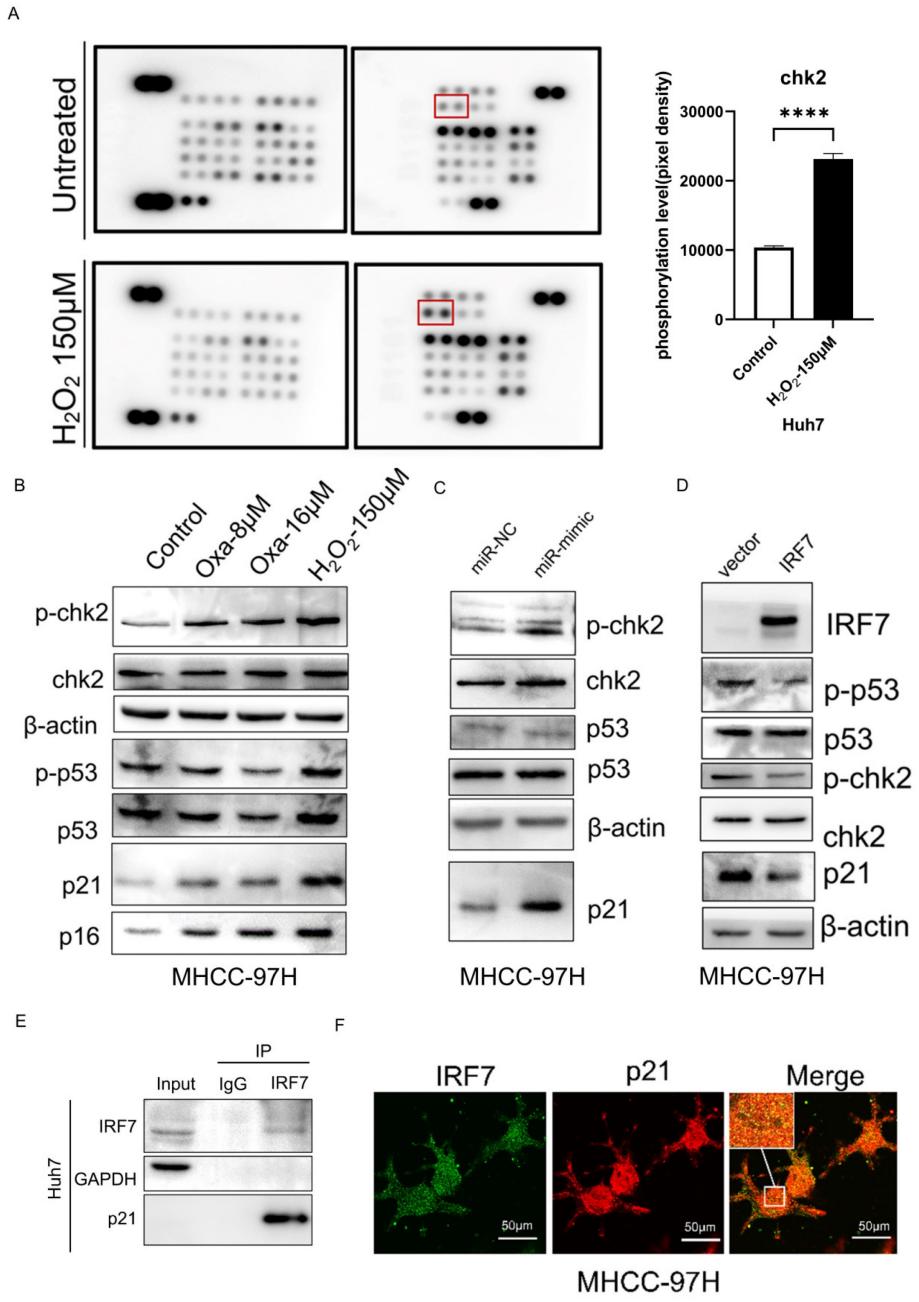
** miRNA-146a-5p induced senescence in HCC cells by upregulating members of the CHK2 signaling pathway.** A. Phosphate kinase antibody chip detection following 150μM H_2_O_2_ pretreatment. Huh7 cell protein lysates that were not pretreated with H_2_O_2_ (control). Measuring the amount of CHK2 phosphorylation based on the density of array pixels. The red box in (A) highlights the chosen proteins. B. Oxaliplatin and H_2_O_2_-induced phosphorylation of p53 (downstream of CHK2) and CHK2 in aged Huh7 cells was demonstrated by Western blot analysis. C. Western blot analysis demonstrating how exosome transfection with mimic-146a-5p altered the phosphorylation of p53 and CHK2 in MHCC-97H cells. D. Western blot analysis demonstrating overexpression of IRF7 altered the phosphorylation of p53 and CHK2 in MHCC-97H cells. E. Co-immunoprecipitation experiments confirmed the interaction between IRF7 and p21 proteins. F. Immunofluorescence assays validated the co-localization of IRF7 and p21 within the nuclei of liver cancer cells. *P<0.05; **P<0.01; ***P<0.001; ****P<0.0001; ns P>0.05.

To further determine the mechanism by which miRNA-146a-5p promotes HCC cell aging, we performed a phosphate kinase antibody array in normal Huh7 cells and H_2_O_2_-induced aging Huh7 cells. As shown in Figure 8A, among the 43 detected kinases, an increase in the phosphorylation of the CHK2 protein was detected in aging HCC cells. Western blot analysis confirmed the changes in the phosphorylation status of the CHK2 protein and its downstream protein p53 after oxaliplatin- and H_2_O_2_-induced aging, as well as the changes in the protein expression of the aging markers p21 and p16 (Figure 8B). To investigate whether miRNA-146a-5p can promote the phosphorylation of CHK2 in HCC cells, western blotting was performed, and the results showed that the phosphorylation of CHK2 and its downstream protein p53 was increased in aging HCC cells, and the aging marker p21 protein was significantly upregulated (Figure 8C). Protein blotting revealed that after the overexpression of IRF7, the protein levels of phosphorylated CHK2 and p53, as well as the protein level of the aging marker p21, were significantly reduced (Figure 8D). To further validate the relationship between IRF7 and p21, we conducted immunoprecipitation experiments as well as immunofluorescence assays. The immunoprecipitation results showed that the IRF7 protein binds to p21 (Figure 8E), and the results of the immunofluorescence assays also indicated that IRF7 and p21 co-localize in the nucleus (Figure 8F). The above results indicate that miRNA-146a-5p may induce HCC cell cycle arrest and promote HCC aging through the CHK2 pathway.

### miRNA-146a-5p attenuates HCC tumor growth *in vivo*

**Figure 9:**
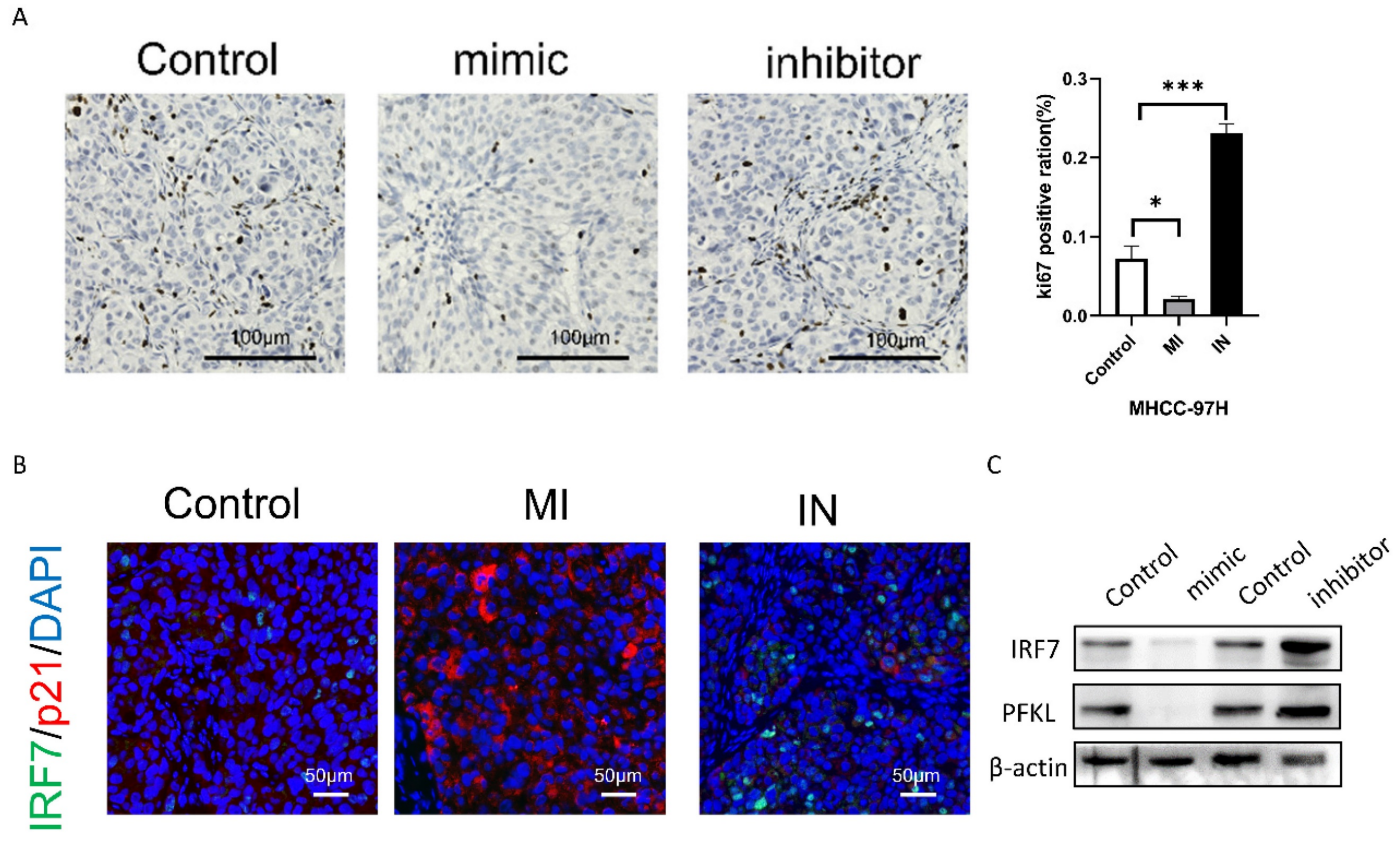
**miR-146a 5p inhibits the growth of HCC cells *in vivo*.** A. The Ki-67 staining results showed that miR-146a 5p inhibited the proliferation of nude mouse liver cancer cells. B. Immunohistochemical staining showed a negative correlation between the expression of IRF7 and miR-146a 5p *in vivo*, while the expression of p21 and miR-146a 5p was positively correlated *in vivo*. C. Western blot analysis showed that IRF7 and PFKL were negatively correlated with the expression of miR-146a 5p *in vivo*. *P<0.05; **P<0.01; ***P<0.001; ****P<0.0001; ns P>0.05.

Next, we will examine the impact of either overexpression or inhibition of miRNA-146a-5p on the proliferation of HCC cells using a subcutaneous xenograft model. By using immunohistochemical analysis, we discovered that the xenograft tumors of MHCC-97H cells overexpressing miRNA-146a-5p showed decreased positive staining for the proliferation marker Ki67 when compared to the xenograft tumors of MHCC-97H cells in the control group (Figure 9B). On the other hand, xenograft tumors made from MHCC-97H cells grew more rapidly when miRNA-146a-5p was inhibited. This was demonstrated by increased tumor weight, volume, and Ki67-positive staining (Figure 9A-B). According to the immunofluorescence labeling results of nude mouse tumors, the expression of p21 and miRNA-146a-5p was positively correlated, the expression of IRF7 and miRNA-146a-5p was negatively correlated, and the expression of p21 and IRF7 was negatively correlated *in vivo* (Figure 9C). Western blotting was used to detect the protein expression levels of IRF7 and PFKL in tumor tissue. Overexpression of miRNA-146a-5p resulted in a significant decrease in IRF7 and PFKL protein expression, while inhibition of miRNA-146a-5p resulted in a significant increase in IRF7 and PFKL protein expression. This once again confirmed that IRF7 is the target gene of miRNA-146a-5p and that miRNA-146a-5p expression is negatively correlated with PFKL protein expression (Figure 9D). In summary, the above data indicate that miRNA-146a-5p induces MHCC-97H cell aging by targeting IRF7 and inhibits glycolysis, which acts as a tumor suppressor in HCC.

## Discussion

Cellular senescence is a process by which cell proliferation is arrested after exposure to various stimuli; this process has been known to have antitumor effects^13^. Moreover, miRNAs are small noncoding RNAs that act as oncogenes or tumor suppressors in different types of cancer^14^. Many miRNAs have been confirmed to be related to aging^15^, such as miR-145, which specifically targets ZEB2 to inhibit the aging of hepatic stellate cells^16^; and miR-21, which can reduce aging-related renal fibrosis^17^.

In this study, the overexpression of miRNA-146a-5p was confirmed in oxaliplatin chemotherapy- and oxidative stress-induced HCC cell aging models. We observed that miRNA-146a-5p is overexpressed in aging liver cancer cells and exosomes. We also found that overexpression of miRNA-146a-5p can promote cell aging and inhibit glycolytic function, indicating that it may be driving liver cancer cell aging by inhibiting energy metabolism. Mechanistically, we found that the overexpression of miRNA-146a-5p reduces cell viability, blocks the cell cycle, induces cell aging, and inhibits glycolytic function. In addition, we showed that miRNA-146a-5p promotes HCC cell aging by upregulating the CHK2 signaling pathway. Our experiments confirmed that one of the target genes of miRNA-146a-5p, IRF7, promotes glycolysis and inhibits HCC cell aging. Moreover, we found for the first time that IRF7 is highly expressed in HCC tissue, and animal experiments confirmed that the expression level of IRF7 is closely related to tumor progression.

In recent years, increasing evidence has shown that IRF7 plays an important role in the immune response, antiviral immunity, and autoimmune diseases^18^. IRF7 induces an immune response by regulating the production of interferon^18^,^19^. IRF7 plays an important role in viral infection^20^. When cells perceive virus invasion, IRF7 is activated and migrates to the nucleus, where it promotes the production of interferon and initiates an antiviral immune response^18^,^19^. In addition to its response to viral infections, IRF7 also participates in regulating other immune processes, including its response to pathogens such as bacteria and fungi^20^. It has been demonstrated that IRF7 is crucial for the development and spread of tumors in a number of cancer types^19^. For instance, genetic silencing of IRF7 dramatically increased bone metastases of breast cancer through immune escape in a mouse model of spontaneous bone metastasis^21^. IRF7 knockdown enhanced lung cancer cells' susceptibility to oncolytic viruses^20^. Furthermore, granulocyte bone marrow-derived suppressor cells (G-MDSCs) were significantly overexpanded in mice with IRF7 loss^22^,^23^, which encouraged the growth and spread of tumors. Low expression of IRF7 in osteosarcoma cells prevents the growth of tumors and the Warburg effect by transcriptionally inhibiting PKM2^23^. Relevant studies on the connection between IRF7 and liver cancer is currently lacking, though.

Based on our research indicating that IRF7 is overexpressed in HCC tissue and that its high expression may be closely related to HCC metastasis and invasiveness, we investigated its potential regulatory effects on glycolytic enzymes and aging, considering the importance of the Warburg effect and aging as markers of tumor metabolism in tumor occurrence and development. According to reports, glycolysis is one of the main pathways by which cells obtain energy, and it plays a crucial role in the energy metabolism of cells^24^. As cells age, their energy metabolism efficiency may decrease, leading to a decrease in glycolytic capacity^25^. During glycolysis, reactive oxygen species (ROS) are produced, and excessive ROS can lead to oxidative stress, damaging cellular structures such as DNA, proteins, and lipids^26^. As age increases, cells may find it more difficult to effectively clear these ROS, thereby accelerating the aging process^4^,^5^,^27^.

Glycolysis and mitochondrial respiration are the two main methods of energy production in cells^28^. As aging progresses, mitochondrial function may decrease, leading to a greater dependence on glycolysis, which may be related to changes in cellular function during the aging process^29^. According to our Seahorse assay results, the overexpression of miRNA-146a-5p leads to the aging of HCC cells, resulting in a decrease in mitochondrial function, basal respiration and ATP production, as well as a decrease in the glycolytic rate. This confirms that as cells age, mitochondrial function decreases, and mitochondria are unable to effectively utilize glucose, leading to a decrease in glycolytic function. In contrast, when the expression level of miRNA-146a-5p is inhibited, the aging of HCC cells decreases, mitochondrial function is promoted, basal respiration and ATP production both increase, and the glycolysis rate increases. This finding confirmed that miRNA-146a-5p can indeed reduce the glycolysis rate of HCC cell lines and inhibit the Warburg effect. Overexpression of IRF7 inhibits cell aging, promotes mitochondrial function, increases basal respiration and ATP production, increases the glycolysis rate, and promotes the Warburg effect. In summary, miRNA products produced during the aging process can affect the metabolic status and aging process of cells, thereby inhibiting the progression of liver cancer.

Threonine/threonine kinase CHK2 is a key component of DNA damage response^30^. In human cells, after genetic toxicity stress, CHK2 is activated and phosphorylated by more than 20 proteins to induce appropriate cellular responses, which depend on the degree of damage, cell type, and other factors, and can be cell cycle checkpoint activation, induction of ternary copolymers or aging, DNA repair, or damage tolerance^31^. CHK2 can stabilize and activate p53 by phosphorylating downstream protein p53. Activated p53 upregulates the expression of p21 (inhibitor of cyclin dependent kinase), leading to G1/S conversion arrest and promoting cell aging^32^. Our experimental results indicate that phosphorylation of CHK2 protein and its downstream protein p53 protein can be detected in aging liver cancer cells, and p21 protein is also significantly increased. This phenomenon was detected in co-culture of miR-146a-5p exosomes with HCC cells or silencing of IRF7, indicating that the mechanism of miR-146a-5p promoting HCC cell aging may be caused by targeting IRF7 to activate the CHK2/p53/p21 pathway.

## Conclusion

This study used a series of experimental methods to clarify the over-expression of miRNA-146a-5p in the whole blood and plasma exosomes of liver cancer patients treated with chemotherapy drugs. *In vitro* and *in vivo* experiments confirmed that miRNA-146a-5p can act as an EV-SASP to inhibit cancer, promote liver cancer cell aging, inhibit aerobic glycolysis of liver cancer cells, and thus achieve the effect of inhibiting liver cancer cell proliferation, migration, and invasion. At the same time, we found that one of the targets of miRNA-146a-5p in the aging pathway, IRF7, can directly bind to the glycolytic enzyme PFKL to promote aerobic glycolysis in liver cancer, and inhibiting IRF7 can also significantly inhibit aerobic glycolysis in liver cancer. And we also found that IRF7 is highly expressed in liver cancer tissue, and high expression of IRF7 can promote liver cancer cell proliferation, migration, and invasion, and IRF7 can inhibit liver cancer aging through CHK2/p53/p21 pathway.

## Supplementary Material

Supplementary tables.

## References

[1] Llovet J M (2021). Hepatocellular carcinoma. Nat Rev Dis Primers.

[2] Yang JD, Hainaut P, Gores GJ, Amadou A, Plymoth A, Roberts LR (2019). A global view of hepatocellular carcinoma: trends, risk, prevention and management. Nature Reviews Gastroenterology & Hepatology.

[3] Yang JD, Heimbach JK (2020). New advances in the diagnosis and management of hepatocellular carcinoma. BMJ.

[4] Calcinotto A, Kohli J, Zagato E, Pellegrini L, Demaria M, Alimonti A (2019). Cellular Senescence: Aging, Cancer, and Injury. Physiol Rev.

[5] Lee S, Schmitt CA (2019). The dynamic nature of senescence in cancer. Nat Cell Biol.

[6] He S, Sharpless NE (2017). Senescence in Health and Disease. Cell.

[7] Kowald A, Passos JF, Kirkwood TBL (2020). On the evolution of cellular senescence. Aging Cell.

[8] Wang C, Vegna S, Jin H, Benedict B, Lieftink C, Ramirez C, de Oliveira RL, Morris B, Gadiot J, Wang W, du Chatinier A, Wang L, Gao D, Evers B, Jin G, Xue Z, Schepers A, Jochems F, Sanchez AM, Mainardi S, Te Riele H, Beijersbergen RL, Qin W, Akkari L, Bernards R (2019). Inducing and exploiting vulnerabilities for the treatment of liver cancer. Nature.

[9] Xu S, Wu W, Huang H, Huang R, Xie L, Su A, Liu S, Zheng R, Yuan Y, Zheng HL, Sun X, Xiong XD, Liu X (2019). The p53/miRNAs/Ccna2 pathway serves as a novel regulator of cellular senescence: Complement of the canonical p53/p21 pathway. Aging Cell.

[10] Thakur A, Parra DC, Motallebnejad P, Brocchi M, Chen HJ (2022). Exosomes: Small vesicles with big roles in cancer, vaccine development, and therapeutics. Bioactive Materials.

[11] Su Y, Li Y, Guo R, Zhao J, Chi W, Lai H, Wang J, Wang Z, Li L, Sang Y, Hou J, Xue J, Shao Z, Chi Y, Huang S, Wu J Plasma extracellular vesicle long RNA profiles in the diagnosis, prediction of treatment response for breast cancer npj Breast Cancer. 2021;7(1).

[12] Ghafouri-Fard S, Abak A, Talebi SF, Shoorei H, Branicki W, Taheri M, Akbari Dilmaghani N (2021). Role of miRNA and lncRNAs in organ fibrosis and aging. Biomed Pharmacother.

[13] Courtois-Cox S, Jones SL, Cichowski K (2008). Many roads lead to oncogene-induced senescence. Oncogene.

[14] Ugalde AP, Ramsay AJ, de la Rosa J, Varela I, Marino G, Cadinanos J, Lu J, Freije JM, Lopez-Otin C (2011). Aging and chronic DNA damage response activate a regulatory pathway involving miR-29 and p53. EMBO J.

[15] Kadota T, Fujita Y, Yoshioka Y, Araya J, Kuwano K, Ochiya T (2018). Emerging role of extracellular vesicles as a senescence-associated secretory phenotype: Insights into the pathophysiology of lung diseases. Mol Aspects Med.

[16] Yang J, Lu Y, Yang P, Chen Q, Wang Y, Ding Q, Xu T, Li X, Li C, Huang C, Meng X, Li J, Zhang L, Wang X (2019). MicroRNA-145 induces the senescence of activated hepatic stellate cells through the activation of p53 pathway by ZEB2. J Cell Physiol.

[17] Zhao S, Li W, Yu W, Rao T, Li H, Ruan Y, Yuan R, Li C, Ning J, Li S, Chen W, Cheng F, Zhou X (2021). Exosomal miR-21 from tubular cells contributes to renal fibrosis by activating fibroblasts via targeting PTEN in obstructed kidneys. Theranostics.

[18] Ma W, Huang G, Wang Z, Wang L, Gao Q (2023). IRF7: role and regulation in immunity and autoimmunity. Front Immunol.

[19] Qing F, Liu Z (2023). Interferon regulatory factor 7 in inflammation, cancer and infection. Front Immunol.

[20] Qin Z, Fang X, Sun W, Ma Z, Dai T, Wang S, Zong Z, Huang H, Ru H, Lu H, Yang B, Lin S, Zhou F, Zhang L (2022). Deactylation by SIRT1 enables liquid-liquid phase separation of IRF3/IRF7 in innate antiviral immunity. Nat Immunol.

[21] Li Y, Huang R, Wang L, Hao J, Zhang Q, Ling R, Yun J (2015). microRNA-762 promotes breast cancer cell proliferation and invasion by targeting IRF7 expression. Cell Proliferation.

[22] Yang Q, Li X, Chen H, Cao Y, Xiao Q, He Y, Wei J, Zhou J (2017). IRF7 regulates the development of granulocytic myeloid-derived suppressor cells through S100A9 transrepression in cancer. Oncogene.

[23] Li Z, Geng M, Ye X, Ji Y, Li Y, Zhang X, Xu W (2022). IRF7 inhibits the Warburg effect via transcriptional suppression of PKM2 in osteosarcoma. International Journal of Biological Sciences.

[24] Payen VL, Porporato PE, Baselet B, Sonveaux P (2016). Metabolic changes associated with tumor metastasis, part 1: tumor pH, glycolysis and the pentose phosphate pathway. Cell Mol Life Sci.

[25] Teoh ST, Lunt SY (2018). Metabolism in cancer metastasis: bioenergetics, biosynthesis, and beyond. Wiley Interdiscip Rev Syst Biol Med.

[26] Ganapathy-Kanniappan S, Geschwind J-FH (2013). Tumor glycolysis as a target for cancer therapy: progress and prospects. Mol Cancer.

[27] Ben-Porath I, Weinberg RA (2005). The signals and pathways activating cellular senescence. Int J Biochem Cell Biol.

[28] Ganapathy-Kanniappan S (2017). Linking tumor glycolysis and immune evasion in cancer: Emerging concepts and therapeutic opportunities. Biochim Biophys Acta Rev Cancer.

[29] Koppenol WH, Bounds PL, Dang CV (2011). Otto Warburg's contributions to current concepts of cancer metabolism. Nature Reviews Cancer.

[30] Zannini L, Delia D, Buscemi G (2014). CHK2 kinase in the DNA damage response and beyond. J Mol Cell Biol.

[31] Di Micco R, Fumagalli M, Cicalese A, Piccinin S, Gasparini P, Luise C, Schurra C, Garre M, Nuciforo PG, Bensimon A, Maestro R, Pelicci PG, d'Adda di Fagagna F (2006). Oncogene-induced senescence is a DNA damage response triggered by DNA hyper-replication. Nature.

[32] Song S, Shi Y, Wu W, Wu H, Chang L, Peng P, Zhang L, Fan J, Gu J, Ruan Y (2021). Reticulon 3-mediated Chk2/p53 activation suppresses hepatocellular carcinogenesis and is blocked by hepatitis B virus. Gut.

