# Supplementary material for: Exosomal miRNA-146a-5p Derived from Senescent Hepatocellular Carcinoma Cells Promotes Aging and Inhibits Aerobic Glycolysis in Liver Cells via Targeting IRF7
Source: J Cancer. 2024 Jun 17;15(14):4448–66. doi: 10.7150/jca.96500 (PMC11242348; doi:10.7150/jca.96500)
Supplement: Supplementary file 1 — Supplementary tables. [file jcav15p4448s1.pdf]

Supplementary Table S1. The primer sequences

	mRNA primers		
**Gene name**	**Species**	**Direction**	**Sequence**
IRF7	Human	FORWARD	GCTGGACGTGACCATCATGTA
		REVERSE	GGGCCGTATAGGAACGTGC
GAPDH	Human	FORWARD	GGAGCGAGATCCCTCCAAAAT
		REVERS	GGCTGTTGTCATACTTCTCATGG
ACTB	Human	FORWARD	CATGTACGTTGCTATCCAGGC
		REVERSE	CTCCTTAATGTCACGCACGAT
HK1	Human	FORWARD	AGGAAGCAGACGCACAACAATG
		REVERSE	ACCAGCAGCACACGGAAATTG
HK2	Human	FORWARD	ACGAGAGCATCCTCCTCAAGTG
		REVERSE	CACCACAGCAACCACATCCAG
PFKL	Human	FORWARD	CACAGGTGCCAACATCTTCCG
		REVERSE	GTGCCGCAGAAGTCGTTATCG
ALDOA	Human	FORWARD	ACCGAGAACACCGAGGAGAAC
		REVERSE	ATCATCCGCCTTCTGGTAGAGTG
PGK1	Human	FORWARD	AGCCAAGTCGGTAGTCCTTATGAG
		REVERSE	TACACAGTCCTTCAAGAACAGAACATC
ENO1	Human	FORWARD	CTTCATCGCTGACCTGGTTGTG
		REVERSE	TCTGAGGAGCTGGTTGTACTTGG
LDHA	Human	FORWARD	GATTCAGCCCGATTCCGTTACC
		REVERSE	AGAGACACCAGCAACATTCATTCC
PFKP	Human	FORWARD	GCTTGCGTCGTGTCACTGAAC
		REVERSE	CATCTTGAAATCTCCTCTCGTCCATC
TPI1	Human	FORWARD	CTCAGAGCACCCGTATCATTTATGG
		REVERSE	GAAGCACCACCCACAAGGAAG
LDHA	Human	FORWARD	GATTCAGCCCGATTCCGTTACC
		REVERSE	AGAGACACCAGCAACATTCATTCC
p21	Human	FORWARD	TGTCCGTCAGAACCCATGC
		REVERSE	AAAGTCGAAGTTCCATCGCTC

	miRNA primers (Tailing Reaction)		
**name**	**Species**	**Direction**	**Sequence**
hsa-miRNA-146-5p	Human	FORWARD	AGCTGGACTGAGAACTGAATTCC

Supplementary Table S2. The specific antibodies

Antibody name	Brand	Nation
anti-GAPDH	Cell Signaling Technology	USA
anti-HSP90	Santa Cruz	USA

anti-Alix	Cell Signaling Technology	USA
anti-Tsg101	ABclonal Technology	China
anti-CD63	Santa Cruz	USA
anti-IgG second antibodies	Cell Signaling Technology	USA
anti-Rabbit second antibodies	Cell Signaling Technology	USA
anti-Mouse second antibodies	Cell Signaling Technology	USA
Alexa Fluor-labeled antibodies	Cell Signaling Technology	USA
anti-IRF7	Proteintech	China
anti-PFKL	Proteintech	China
anti-p21	Abcam	USA
anti-p53	Proteintech	China
anti-p16	Abcam	USA
anti-CHK2	Abcam	USA
anti-CHK2（phospho T68）	Abcam	USA
p53(Phospho-Ser315)	Signalway antibody	USA
